# Failure to Detect XMRV-Specific Antibodies in the Plasma of CFS Patients Using Highly Sensitive Chemiluminescence Immunoassays

**DOI:** 10.1155/2011/854540

**Published:** 2011-07-27

**Authors:** Brendan Oakes, Xiaoxing Qiu, Susan Levine, John Hackett, Brigitte T. Huber

**Affiliations:** ^1^Pathology Department, Tufts University School of Medicine, 150 Harrison Avenue, Boston, MA 02111, USA; ^2^Pharmacology Program, Tufts University School of Medicine, 150 Harrison Avenue, Boston, MA 02111, USA; ^3^Infectious Diseases R&D, Abbott Diagnostics, 100 Abbott Park Road, Abbott Park, IL 60064, USA; ^4^Private Practice, 115 East 72nd Street, New York, NY 10021, USA

## Abstract

In 2009, Lombardi et al. reported their startling finding that the gammaretrovirus xenotropic murine leukemia virus-related retrovirus (XMRV) is present in 67% of blood samples of patients suffering from chronic fatigue syndrome (CFS), as opposed to only 3.7% of samples from healthy individuals. However, we and others could not confirm these results, using a nested PCR assay. An alternative to this highly sensitive, but contamination-prone, technique is to measure the serological response to XMRV. Thus, we tested the plasma samples from our cohorts of CFS patients and healthy controls for the presence of XMRV-specific antibodies. Using two novel chemiluminescence immunoassays (CMIAs), we show that none of our samples have any XMRV-reactive antibodies. Taken together with our previous findings, we conclude that XMRV is not present in any human individual tested by us, regardless of CFS or healthy control.

## 1. Introduction

In 2006, Urisman et al. identified a new gammaretrovirus in prostate cancer samples harboring a mutation in a viral defense gene known as *RNASEL *[1]. This new virus, xenotropic murine leukemia virus-related retrovirus (XMRV), was found to be a close relative to known murine leukemia viruses (MLVs) and was the first documented case of human infection with a xenotropic retrovirus. Although XMRV was originally associated with the mutant variant of the *RNASEL *gene, further research could not confirm this association but did find it in about 10% of prostate cancers [2]. 

The discovery of a new virus that could infect humans lead Lombardi et al. [3] to test for the virus in patients suffering from chronic fatigue syndrome (CFS). CFS is a disease of unknown etiology that manifests as neurological, immunological, and endocrinological dysfunctions. A wide range of viruses have been investigated in the past as causative agents of CFS; however, findings were mixed, and no conclusive evidence of one virus causing CFS has been implicated [4]. Using a nested polymerase chain reaction (PCR), Lombardi et al. found that blood samples of 68 out of 101 (67%) CFS patients contained the XMRV *gag* sequence, as opposed to only 8 out of 212 (3.7%) samples from healthy individuals [3]. The finding of a virus linked to CFS reignited excitement in the field, leading many laboratories around the world to test for this new virus, but the excitement has been short lived. Although some support linking XMRV or MLVs and CFS has been published [3, 5, 6], it has been overshadowed by reports failing to detect the virus in CFS patients [7–20], including a study done by us. 

In our original paper [17], we failed to find an association between CFS patients and XMRV, using PCR technology. However, we did detect some XMRV sequences as well as other MLV sequences in some of our samples. Due to the close relationship between XMRV and MLVs, which are present throughout the mouse genome, we tested all of our samples for mouse DNA using a TaqMan qPCR assay for murine mitochondrial cytochrome oxidase, *cox2 *[14], as well as a single PCR assay for the highly abundant intracisternal A-type particle (IAP) long terminal repeat sequence, developed by our group [17]. We found that every sample that contained an XMRV or MLV sequence was also positive for mouse DNA contamination. Although we did not claim that our findings provided a full explanation of the origin of XMRV, we put forward a cautionary tale about the risks of mouse DNA contamination in various common laboratory reagents. 

One of the criticisms of our study [17] was that we only used PCR technology to test for the presence of XMRV, while the original paper also included serological analyses [3]. Specifically, some groups have developed novel serological tests utilizing western blots and ELISAs in the search for anti-XMRV antibodies, because the presence of antibodies could not be due to mouse DNA contamination [3, 8, 13, 14, 20, 21]. Recently, two prototype direct format chemiluminescent immunoassays (CMIAs) were developed to detect XMRV-specific antibodies [22]. Both CMIAs utilize a direct assay format in which recombinant p15E or gp70 protein serves as both capture and detection antigens. The assays demonstrated excellent sensitivity, detecting early seroconversion bleeds in XMRV-infected rhesus macaques [22]. Moreover, these assays were also shown to detect specific antibodies to MLVs [22]. In this study, we use these two sensitive CMIAs to screen plasma samples from our blinded cohorts for the presence of XMRV-specific antibodies. No samples from our cohort of over 100 CFS patients were positive in either of these assays, while two samples from the healthy control cohort tested positive in one of the CMIA assays; however, reactivity of these same samples was not confirmed by western blot. Thus, these highly sensitive serological studies have confirmed our prior conclusion that the positive XMRV PCR results were a result of mouse DNA contamination, since no antibodies against XMRV were present.

## 2. Materials and Methods

### 2.1. Sample Collection

All samples were collected according to the institutional guidelines of Tufts University, after receiving informed consent. The 36 healthy individuals (15 females and 21 males) were recruited on a voluntary basis by the Huber laboratory and were between 18 and 65 years of age. The 112 CFS patients (90 females, 20 males, and 3 unknown), recruited by Dr. Susan Levine, were between 18 and 65 years of age and resided in the Northeastern United States. All patients were diagnosed for CFS according to the CDC criteria, and the majority was completely disabled. The cohort comprised a combination of those with an abrupt and others with a gradual onset of symptoms.

### 2.2. Preparation of Human Blood and Plasma Samples

Approximately 30 mL of blood were drawn into three heparinized tubes (Becton Dickinson) and shipped overnight (CFS patients) or processed immediately (healthy controls). The blood collection tubes from each individual were consolidated into one 50 mL tube and diluted with PBS, containing CaCl2 and MgCl2 (sigma) at a 1 : 1 ratio. 15 mL of Ficoll (GE Healthcare) was added to two new 50 mL tubes, and 25 mL of the diluted blood was gently layered on top of the Ficoll, followed by a 30 min centrifugation in a Sorvall RT7 plus rotor at 2000 rpm at room temperature. The PBMCs were collected from the interface following the spin and were used for DNA isolation. Ten mL of plasma were also collected from each sample and stored at −80°C. One ml of plasma was sent to Abbott Labs on dry ice overnight for further testing.

### 2.3. XMRV Chemiluminescent Immunoassays (CMIAs)

A detailed procedure can be seen here [22]. Briefly, 100 *μ*L of neat plasma were screened for antibodies to XMRV gp70 and p15E proteins using two prototype ARCHITECT chemiluminescent immunoassays (CMIAs; Abbott Diagnostics, Abbott Park, Ill). The CMIAs utilize a direct assay format in which *E. coli*-expressed XMRV p15E or mammalian-expressed XMRV gp70 were used as both capture and detection antigens. Assay positive controls were derived from XMRV-infected macaque plasmas at 1 : 1000 (PC1) or 1 : 4000 (PC2). A pool of normal human plasma was used as negative control (NC) and as sample diluents. Cutoff (CO) values of the ARCHITECT CMIAs were calculated based on the following formulas: CO = 0.45 × (Calibrator 1 Mean Relative Light Units (RLU)) for p15E CMIA and CO = 0.078 × (Calibrator 2 Mean RLU) for gp70 CMIA. Assay results were reported as the ratio of the sample RLU to the cutoff RLU (S/CO) for each specimen. Specimens with S/CO values <1.00 were considered nonreactive; specimens with S/CO values ≥1.00 were considered initially reactive. The S/CO values of the NC, PC1, and PC2 were 0.16, 12.8, and 3.5 for the gp70 CMIA and 0.13, 7.4, and 2.2 for the p15E CMIA. Initially reactive specimens were retested in duplicate by either ARCHITECT p15E or gp70 CMIAs. Repeatedly reactive specimens were analyzed at 1 : 100 dilution by investigational western blot assays using purified XMRV viral lysate as well as recombinant gp70 protein.

### 2.4. Western Blot Analysis

Western blot (WB) analysis using purified XMRV viral lysate as well as recombinant gp70 protein was performed as described [22]. Briefly, viral lysate (80 *μ*g/gel) or recombinant gp70 protein (20 *μ*g/gel) were separated by electrophoresis on a 4–12% NuPAGE Bis-Tris 2-dimension gel (Invitrogen, Carlsbad, Calif) in the presence of sodium dodecyl sulfate (SDS). The protein bands on the gel were electrophoretically transferred to a polyvinylidene difluoride (PVDF) membrane (invitrogen). After blocking, the PVDF membrane was cut into 2 mm strips. Strips were incubated with human samples diluted 1 : 100 or XMRV-infected macaque plasma diluted 1 : 200 overnight at 2–8°C. After removal of unbound antibodies, strips were incubated with alkaline phosphatase conjugated goat antihuman IgG (Southern Biotech, Birmingham, Ala) for 30 minutes at room temperature. The strips were washed, and chromogenic substrate solution was added.

## 3. Results

**Figure 1:**
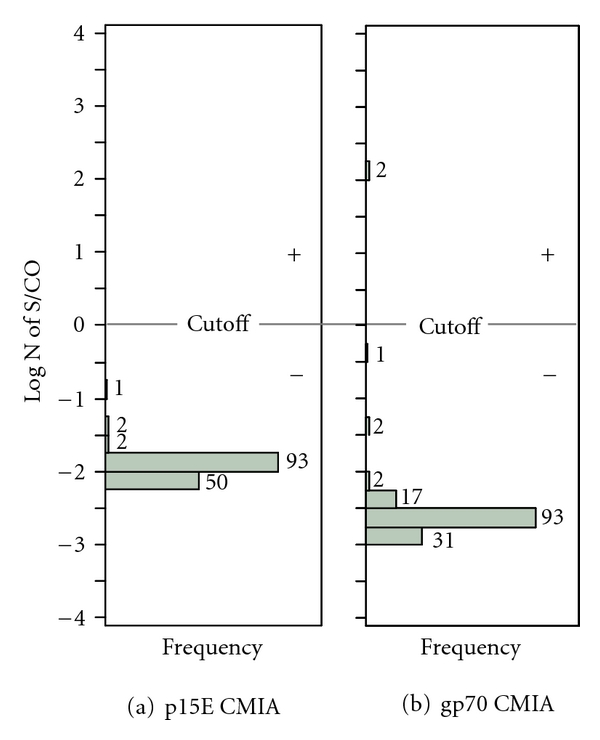
Distribution of p15E CMIA (a) and gp70 CMIA (b) log N of S/CO on 148 samples collected from 112 CFS patients and 36 healthy controls. Numbers of specimens within each log N of S/CO value are shown above the solid bars. Assay cutoffs were equivalent to mean 16 SD and 12 SD for p15E and gp70 CMIAs, respectively, based on blood donor populations [22]. Log N of S/CO, natural log transformation of S/CO.

**Figure 2:**
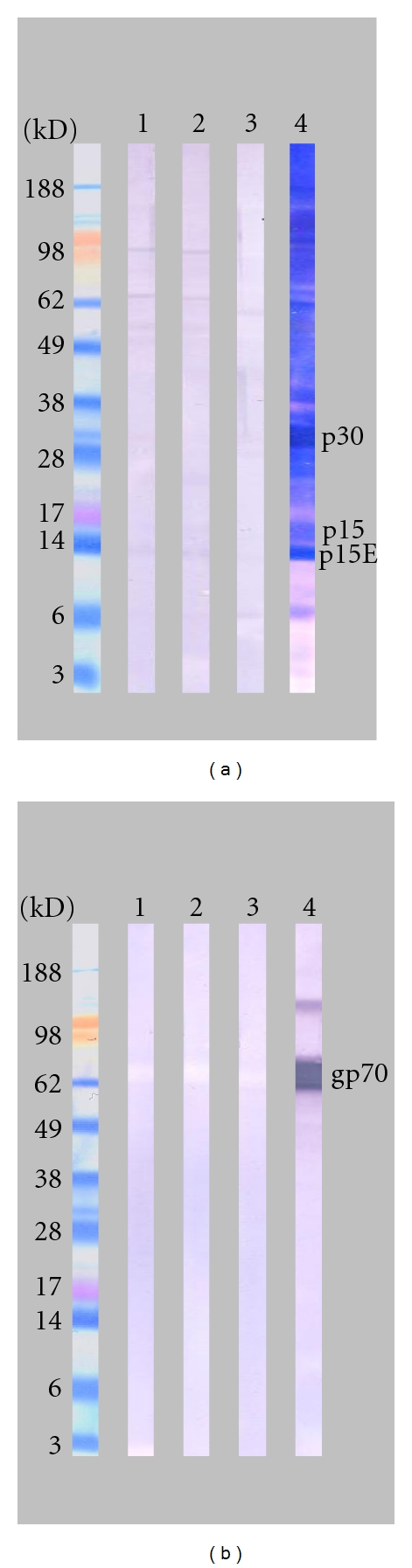
WB analysis of gp70 CMIA reactive samples with (a) native XMRV viral proteins and (b) recombinant gp70. WB strip key: 1 & 2: gp70 reactive samples 137 and 138; 3: normal blood donor plasma as negative control; 4: XMRV-infected macaque plasma as positive control. The faint white band in the 65–70 kd region in (B, strips no. 1–3) indicates a lack of specific anti-gp70 antibody.

148 blinded plasma samples from our original CFS and healthy control cohorts were analyzed for the presence of XMRV-specific antibodies, using the direct format ARCHITECT p15E and gp70 CMIAs. None of the 148 plasma samples were reactive in the p15E CMIA (Figure 1(a)). Two of the 148 samples (ID = 137, 138) were positive in the gp70 CMIA (Figure 1(b)). Both specimens were weakly reactive in the gp70 CMIA with sample/cut-off (S/CO) values of 7.77 (log N of S/CO = 2.05) and 9.02 (log N of S/CO = 2.20), respectively. Although the samples were repeat reactive in the gp70 CMIA, they were not reactive by WB. As shown in Figure 2, both samples showed no visible WB bands using either XMRV viral lysate proteins (Figure 2(a)) or recombinant gp70 protein (Figure 2(b)). Unblinding of the samples revealed that the two gp70 reactive samples stemmed from two sequential blood collections of a single healthy control (Table 1). 

## 4. Discussion

In our original study, we found no specific relationship between the presence of XMRV and CFS [17]. However, screening the genomic DNA from peripheral blood lymphocytes of both healthy control and CFS cohorts, we did detect PCR products that were identical to XMRV *gag *sequences, as well as other MLV *gag* sequences. Due to the high number of MLV sequences in the mouse genomic DNA, we found it prudent to test for mouse DNA contamination in our samples. Using both a test developed by the Switzer lab at CDC for mouse mitochondrial DNA [14], as well as a test developed by the Coffin lab for the IAP [17], we found that every sample that was positive for XMRV or other MLVs PCR products was also positive for mouse DNA. Although these data provide an explanation for the detection of MLV sequences in our samples, they do not rule out the possibility that XMRV and mouse DNA contamination could be present in the same sample. To clarify this issue, we tested our plasma samples for the presence of XMRV-specific antibodies.

Recent animal studies showed that XMRV infection elicited a potent humoral immune response in rhesus macaques [22]. The infected macaques developed XMRV-specific antibodies within two weeks of infection and persisted more than 158 days. The predominant responses were to all three structural proteins of XMRV: the envelope protein gp70, the transmembrane protein p15E, and the capsid protein p30 [22]. Sensitivity of both p15E and gp70 CMIAs was validated by the animal model; both CMIAs were able to detect p15E or gp70 specific antibodies as early as day 9 after infection [22]. In contrast, we were unable to detect XMRV p15E or gp70 specific antibodies in the 112 CFS patients and the 36 healthy controls. Although 2 samples from the same healthy control had weak reactivity in gp70 CMIA, the reactivity was not confirmed by recombinant gp70 WB. Furthermore, both samples were nonreactive in p15E CMIA and had no detectable p15E and p30 antibodies by viral lysate WB. Considered in combination with the negative PCR data, the observed isolated and weak gp70 reactivity most likely represents nonspecific reactivity since specificity of the gp70 CMIA was reported as 99.5% [22]. In summary, the serologic data obtained in this study suggests a lack of XMRV infection in our CFS patients and healthy controls. It is theoretically possible that XMRV replicates at very low levels in humans and fails to induce a humoral immune response, or, alternatively, that it is sequestered or latent and specific antibody titers have declined to undetectable levels over time. Although these possibilities cannot be formally excluded, they seem unlikely given responses observed to other human retroviruses. The combination of negative molecular and serologic data do not support an association between CFS and XMRV or other MLVs. Furthermore, the recent demonstration that XMRV is a recombinant of two murine MLVs (23) raises doubts about the validity (24) of the original XMRV claims in CFS (3).

## 5. Conclusion

With the serological data added to our original finding, we can unequivocally conclude that XMRV is not present in our CFS patient or healthy control cohort samples. Although we have detected XMRV *gag *sequences in three of our samples, they all tested positive for mouse DNA and tested negative for XMRV-specific antibodies. Laboratory mouse strains, as well as wild mice, all carry numerous endogenous MLVs, and extreme caution must be taken when testing for murine-related viruses.

## Figures and Tables

**Table 1:** Results summary for XMRV positive PCR samples. All samples that tested positive for XMRV *gag* sequence in original study [17], as well as the two samples that reacted with the gp70 CMIA, are displayed. Bolded samples showed the VP42 *gag* sequence but did not react with the CMIAs. The italic data shows the two samples that were reactive in the gp70 CMIA. CMIA values less than one are considered nonreactive. XMRV GAG: Nested gag PCR. Mcox: murine mitochondrial cytochrome oxidase qPCR. IAP: Intracisternal A-type particle PCR.

					Initial	Initial	Repeat
		PCR results			test	test	test
							
		XMRV			p15E	gp70	gp70
ID	Unblinded ID	GAG	Mcox	IAP	S/CO	S/CO	S/CO
72	TH72.1	+	+	+	0.38	0.06	
128	TH04.1	+	+	+	0.16	0.07	
129	TH01.7	+	+	+	0.15	0.06	
131	TH01.8	+	−	+	0.12	0.06	
132	TH01.3	+	+	+	0.15	0.06	
**134**	**TH06.1**	**+**	**+**	**+**	**0.15**	**0.07**	
135	TH01.1	+	+	+	0.14	0.09	
136	TH05.1	+	+	+	0.16	0.06	
***137***	***TH07.1***	***+***	***+***	***+***	***0.16***	***7.77***	***7.17, 7.21***
***138***	***TH07.2***	*−*	*−*	*−*	***0.14***	***9.02***	***8.65, 8.77***
143	TH10.1	+	+	+	0.14	0.07	
144	TH11.1	+	−	+	0.14	0.06	
147	TH02.1	+	+	+	0.14	0.07	
152	TH01.5	+	+	+	0.13	0.07	
153	TH21.1	+	+	+	0.15	0.07	
155	TH20.1	+	+	+	0.16	0.06	
156	TH02.2	+	+	+	0.17	0.07	
**158**	**TH08.1**	**+**	**+**	**+**	**0.13**	**0.07**	
**160**	**TH03.1**	**+**	**+**	**+**	**0.13**	**0.07**	
161	TH12.1	+	+	+	0.11	0.06	
163	TH19.1	+	+	+	0.16	0.72	0.75, 0.72
164	TH16.1	+	+	+	0.15	0.07	
